# A pan-cancer analysis for the oncogenic role of cyclin-dependent kinase inhibitor 1B in human cancers

**DOI:** 10.1007/s12672-023-00746-8

**Published:** 2023-07-11

**Authors:** Hao Huang, Duoliang Qiu, Zhengyang Zhou, Biaobiao Wu, Lening Shao, Yuwei Pu, Tengfei He, Yongyou Wu, Dawei Cui, Fengyun Zhong

**Affiliations:** 1 Department of General Surgery, The Second Affiliated Hospital of Soochow University, Suzhou, China; 2 The First Affiliated Hospital, Zhejiang University School of Medicine, Hangzhou, China

**Keywords:** CDKN1B, Pan-cancer, Prognosis, Cyclin-dependent kinase, Gene

## Abstract

**Background:**

Human health and life are threatened by cancer with high morbidity and mortality worldwide. In many experiments, CDKN1B level is associated with cancer risk, Nevertheless, no pan-cancer analysis has been conducted on CDKN1B in human cancers.

**Methods:**

With the help of bioinformatics, a pan-cancer analysis was conducted on the expression levels of CDKN1B in cancer tissues and adjacent tissues from the TCGA, CPTAC and GEO databases. The CDKN1B expression levels in tumor patients was further validated using immunohistochemistry (IHC) and quantitative real-time PCR.

**Results:**

In the study, we first investigated the cancer-related roles of CDKN1B’s in 40 tumors with malignancy. The CDKN1B gene encodes the p27^Kip1^ protein, which can block the production cyclin-dependent kinase (CDK), which is obviously related to the function and survival of cancer cells and alters the prognosis of cancer patients. Furthermore, CDKN1B function requires both protein processing and RNA metabolism. Additionally, the elevated expression of the CDKN1B gene and protein was validated in several cancer tissues from the patients.

**Conclusions:**

These results showed that the levels of CDKN1B were considerably different in a number of cancer tissues, offering a potential future target for cancer therapy.

**Supplementary Information:**

The online version contains supplementary material available at 10.1007/s12672-023-00746-8.

## Introduction

It is of great significance to conduct a pan-cancer analysis of one certain gene and e investigate its association with the clinical prognosis of cancer patients due to the intricacy of carcinogenesis. The largest database of cancer gene at the moment is The Cancer Genome Atlas (TCGA) dataset (https://portal.gdc.cancer.gov/), which is the preferred tool for cancer research due to its enormous sample size, varied data types and standardized information format, among other advantages [1–4]. The Gene Expression Omnibus (GEO) (https://www.ncbi.nlm.nih.gov/geo/), a comprehensive and free functional genomics database, which has around 110,000 datasets with approximately 2.9 million specimens [5]. The two databases were mainly used in this research, which also helped to validate the analysis’ findings [1, 2, 6].

The cyclin-dependent kinase inhibitor 1B (CDKN1B) gene, a cyclin-dependent kinase inhibitor (CDKI) belongs to the kinase inhibitory protein (Kip) family, encodes p27^kip1^ protein [7, 8]. Additionally, the CDKI gene can also comprise the proteins CDKN1A (encoding p21^Waf1^) and CDKN1C (encoding p57^Kip2^). The cyclin- and CDK-binding domains, which are located at the N-terminal sections of the three CDKI proteins, are extremely similar. By combining with cyclin E and CDK2, CDKN1B can prevent the cell cycle from entering G1 to S phase. The cellular transition from a quiescent to a proliferative phase, which is responsible for the degradation of the CDKN1B protein, is driven by the CDK-dependent phosphorylation and subsequent ubiquitination by S-phase kinase-associated protein 1 (SKP1)-cullin 1-F-box protein (SCF) complexes. Therefore, the regulation of CDKN1B has an impact on tumor cells due to its relationship with the cell cycle. Previous studies have analyzed the structure and function of CDKN1B across different diseases [9, 10]. In addition, functional relationships between CDKN1B and carcinogenesis of leukemia, breast cancer, colorectal cancer and other cancers have also been examined in many articles [8, 10–13]. A previous study showed that targeting CDKN1B can induce resistance of head and neck cancer to cisplatin [9]. Furthermore, another study indicated that recombinant adeno-associated virus (rAAV)-based PTEN and CDKN1B delivery contributed to the development of novel therapy for prostate cancer (PCa) [14]. Additionally, a germline deletion of the CDKN1B gene in mice was utilized to validate a mouse model of colorectal cancer resulting from TGF-β-dependent T-cell-specific loss of Smad4 (Smad4^TKO^), which indicated that the loss of CDKN1B was related to aggressive tumor behavior and a poor clinical outcome in cancers [15]. Due to the crucial role of CDKN1B in tumor progression, a pan-cancer analysis of the link between CDKN1B and diverse tumor types is critical for cancer therapy.

In the current study, we first addressed a pan-cancer analysis for the CDKN1B gene utilizing the TCGA and GEO databases in various malignancies. In addition to exploring the underlying mechanisms of CDKN1B in the carcinogenesis and clinical prognosis of malignancies, this investigation additionally examined at gene expression, survival status, immune infiltration and genetic alterations. Furthermore, the presence of CDKN1B in a variety of patient cancer tissues was validated.

## Materials and methods

### Gene expression analysis

In order to analyze the data, we utilized the Tumor Immune Assessment Resource, Version 2 (TIMER2) website (http://timer.cistrome.org/) [16]. In TCGA project, CDKN1B was entered into the “Gene_DE” module, and differences in CDKN1B gene expression level between various malignancies or particular cancer subtypes and adjacent tissues were detected. For certain cancers without normal tissues or with highly restricted normal tissues, the analysis for CDKN1B was performed by the Gene Expression Profiling Interaction Analysis, Version 2 (GEPIA2) website (http://gepia2.cancer-pku.cn/#analysis) [17]. From genotype-tissue expression (GTEx) dataset, boxplots of the variations in CKDN1B gene expression level between these cancer tissues and the normal ones were gathered, with a cutoff of P value = 0.01 and log2FC (fold change) cutoff = 1.

As for the protein expression level of CDKN1B, a crucial interactive web named the University of Alabama at Birmingham Cancer Data Analysis Portal (UALCAN), which can be accessed at http://ualcan.path.uab.edu/analysis-prot.html [18], was performed. This portal was frequently used to analyze and assess protein expression from the Clinical Proteomic Tumor Analysis Consortium (CPTAC) datasets [19, 20]. Entering “CDKN1B” allowed us to compare the total protein levels of CDKN1B in primary tumor tissues and normal tissues. Breast cancer, uterine corpus endometrial carcinoma (UCEC), clear cell renal cell carcinoma (RCC), and lung adenocarcinoma (LUAD) were enrolled in the study.

### Survival prognosis

GEPIA2 is an enhanced web server for large-scale expression profiling and interactive analysis. In addition to gene differential expression analysis, it can also be used for survival analysis. Through GEPIA2’s “Survival Map” module, information on overall survival (OS) and disease-free survival (DFS) linked to CDKN1B expression in all cancers from TCGA was acquired. The thresholds for the high and low expression cohorts were divided using the cut-high (> 50%) and cut-low (< 50%) values, respectively. The “Survival Analysis” module of GEPIA2 looked at survival plots while the long-rank test was used for hypothesis testing.

### Genetic alteration analysis

The cBioPortal website (http://www.cbioportal.org) is an integrated database for retrieving, downloading, analyzing, and visualizing cancer genomics data from a wide range of genomic data types, such as somatic mutations, DNA copy number changes (CNAs), and DNA methylation. The cBioPortal website can perform for a variety of analyses, including the mutation-related analyses and visualization [21]. The “TCGA Pan Cancer Atlas Study” was chosen in the “Quick Selection” section after logging in to the cBioPortal website [22], and “CDKN1B” was entered to search for the gene alteration signature of CDKN1B. The frequencies of alterations, mutation types, and copy number alterations (CNA) data for all TCGA cancers were obtained through the website’s “Cancer Type Summary” module. The CDKN1B mutation location was visible in the three-dimensional (3D) schematic diagram of the protein structure via the website’s “mutation” tool. Additionally, Kaplan–Meier graphs were produced.

### Infiltration analysis

The association between CDKN1B expression and cell infiltration, particularly the cancer-associated fibroblasts (CAFs), was studied across all TCGA malignancies using the TIMER2 web server’s “Immune-Gene” module. The “Immune-Gene” module mainly shows the relationship between tumor gene expression and immune infiltration. To estimate the cell infiltration in cancer tissues, the algorithms XCELL, EPIC, and MCPCOUNTER were employed. The P values and partial correlation (cor) values were calculated with Spearman’s rank correlation test with purity adjustment, and the results were then shown as a heatmap and scatter plot.

### CDKN1B-related gene enrichment analysis

The STRING website (https://string-db.org/) is a database that searches for known and predicted interactions between proteins. On the STRING website, we conducted searches based on the names of proteins in particular (“CDKN1B”) and organisms (“Homo sapiens”) [23, 24]. Next, the key variables were as follows: the lowest minimal interaction score needed [“Low confidence (0.150)”, the significance of network edges (“evidence”), the most interactors to display (“no more than 50 interactors” in the first shell), and the active interaction sources (“experiments”). The experiments eventually yielded the determined CDKN1B-binding protein.

Subsequently, utilizing the “Similar gene detection” module of GEPIA2, we screened the top 100 CDKN1B-related targeted genes based on the datasets from all TCGA tumor and normal tissues. For a pairwise gene between CDKN1B and homologous genes, the “correlation analysis” module was used. Log2TPM was used for generating dot plots. Meanwhile, the heatmap data for the selected genes were obtained by the “Gene_Corr” module of TIMER2.

Jvenn, an interactive Venn diagram viewer, was addressed for the intersection analysis to compare the CDKN1B-binding and interacting genes [25]. The Kyoto Encyclopedia of Genes and Genomes (KEGG) pathway analysis was performed using the two sets of data. Shortly after the genes were uploaded to Database for annotation, visualization, and integrated discovery (DAVID), the species (“Homo sapiens”) and chosen IDs (“OFFICIAL_GENE_SYMBOL”) were acquired for the functional annotation map [26]. Last but not least, the enriched pathways were shown using the “tidyr” and “ggplot2” R packages (http://www.r-project.org/).

### Tissue microarray and immunohistochemical staining

The cancer tissues and their adjacent tissues were obtained from patients enrolled in the Department of Pathology of The Second Affiliated Hospital of Soochow University in Suzhou, China. To develop the tissue microarray (TMA), a 3-mm-diameter core of the tumor tissue area was confirmed. Slides made from TMA were deparaffinized in xylene, rehydrated in ethanol, and the antigens were extracted for immunohistochemistry (IHC) tests. The slides were blocked with goat serum for 1 h, and after washing, the slides were further stained with the diluted anti-anti-p27^Kip1^ (CDKN1B) antibody (Abcam, ab32034). Following the manufacturer’s instructions, 3,3-diaminobenzidine (DAB) was administered to stain the samples after 24 h. IHC results of p27^Kip1^ staining scores were semiquantified by an immunoreactive scoring (IRS) system, which was previously described [27].

### Quantitative real-time PCR

Using the RNeasy Mini Kit from Qiagen (Germany) and a reverse transcription reagent kit from Takara (China), total RNA was isolated from fresh paracancerous tissue (PCT) and cancer tissue (CT) of human stomach adenocarcinoma (STAD), the integrity and concentration of the RNA was calculated by NanoDrop 2000 spectrophotometer (Thermo Scientific, USA), and which was synthesize to cDNA by a reverse transcription reagent kit (Takara, China). The expression levels of target genes were detected in triplicate by Takara SYBR Supermix (Takara, China) using a real-time quantitative polymerase chain reaction (qPCR) assay via BIO-RAD CFX96. As a sample internal control, glyceraldehyde 3-phosphate dehydrogenase (GAPDH) was used. The sequences of the specific primers for the CDKN1B and associated six genes were listed in Table 1, and the relative levels of the target genes were analyzed by the 2^−ΔΔCt^ method.

**Table 1:** Sequences of primers for the qPCR

Gene	Sequence
GAPDH	Forward: 5ʹ-GTCTCCTCTGACTTCAACAGCG-3ʹ
	Reverse: 5ʹ-ACCACCCTGTTGCTGTAGCCAA-3ʹ
CDKN1B	Forward: 5ʹ-ATAAGGAAGCGACCTGCAACCG-3ʹ
	Reverse: 5ʹ-TTCTTGGGCGTCTGCTCCACAG-3ʹ
GPBP1	Forward 5ʹ-CATGAGTCCACATTTGGCGTTGG-3ʹ
	Reverse 5ʹ-CGTGTCAGTTTGGTTAGACGAGG-3ʹ
KIF1BP	Forward 5ʹ-GATCCTACTGAGCGTTTTCTTCC-3ʹ
	Reverse 5ʹ-GGCAATAGTGAGCAGCCTTCTC-3ʹ
NECAP1	Forward 5ʹ-ATTGGACTGGTCGCCTCCGAAT-3ʹ
	Reverse 5ʹ-TAGCGGCTAGAATCTGTCACCG-3ʹ
THAP5	Forward 5ʹ-GACTCTCTTGACATCAGATGGGG-3ʹ
	Reverse 5ʹ-GGCATACTTCTTTCTCATCTTCCA-3ʹ
TRIM23	Forward 5ʹ-GTGAAGCACACAGCGAACTTGC-3ʹ
	Reverse 5ʹ-CTTCTACTGACAGTGCTCCAGC-3ʹ
ZNF263	Forward 5ʹ-AGGTGGGACAAGGAGGAAAGCT-3ʹ
	Reverse 5ʹ-GCATACAGACGGAACACCTTCC-3ʹ

### Statistical analysis

The Graphpad Prism (version 7.0) and R software (version 4.1.1) is used for statistical analysis and mapping. Student’s t-test identified the differences between two groups. P < 0.05 was considered statistically significant.

## Results

### Gene expression analysis

**Fig. 1:**
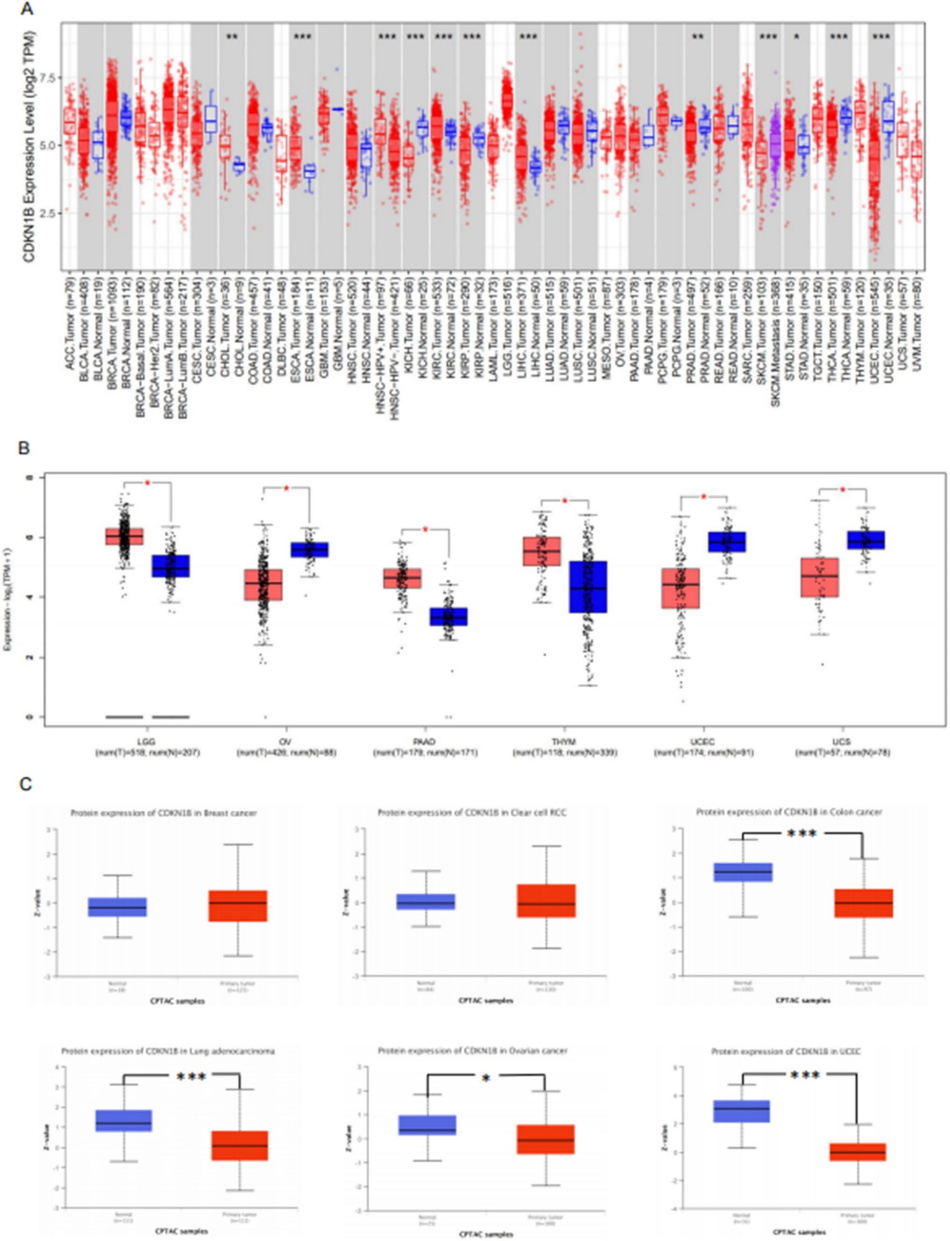
Expression level of CDKN1B in different tumors. **A** The level of the CDKN1B gene in different cancers or specific cancer subtypes was analyzed through TIMER2. **B** The equivalent normal tissues from the GTEx database were used as controls for the types of DLBC, LAML, LGG, OV, SKCM, TGCT, THYM, and UCS in the TCGA experiment. Data for the box plot were provided. **C** The expression levels of CDKN1B total protein in normal tissue and primary tissue from breast cancer, clear cell RCC, colon cancer, lung adenocarcinoma, ovarian cancer, and UCEC were also compared using the CPTAC dataset. *P < 0.05, **P < 0.01, ***P < 0.001

The TIMER2 assay was performed to calculate the levels of CDKN1B gene in 40 cancer types from the TCGA database in order to investigate the oncogenic consequences of human CDKN1B. The expressions of the CDKN1B gene were significantly elevated in the tumor tissues of cholangiocarcinoma (CHOL), esophageal carcinoma (ESCA), head and neck squamous cell carcinoma–human papillomavirus± (HNSC–HPV±), kidney renal clear cell carcinoma (KIRC), hepatocellular carcinoma (HCC), and STAD, as displayed in Fig. 1A. However, in several tumor tissues from kidney chromophobe (KICH), kidney renal papillary cell carcinoma (KIRP), prostate adenocarcinoma (PRAD), skin cutaneous melanoma (SKCM), thyroid carcinoma (THCA), and UCEC, the level of CDKN1B was noticeably decreased, respectively. As controls, we utilized normal tissue from the GTEx dataset, and we further evaluated the difference in CDKN1B expression between the controls and cancer tissues from the brain lower grade glioma (LGG), ovarian serous cystadenocarcinoma (OV), thymoma (THYM), and uterine carcinosarcoma (UCS) (Fig. 1B). However, there was no statistically significant difference in CDKN1B expression between the cancer tissues from diffuse large B-cell lymphoma (DLBC), acute myeloid leukemia (AML), SKCM, and testicular germ cell tumors (TGCT), all of which are lymphoid neoplasms (Fig. 1B).

Based to the CPTAC dataset, CDKN1B protein levels were reduced in tumor tissues from breast cancer and clear cell RCC compared to controls (Fig. 1C), but they did not statistically differ from colon cancer, lung adenocarcinoma, ovarian cancer, and UCEC, respectively.

### Survival analysis

**Fig. 2:**
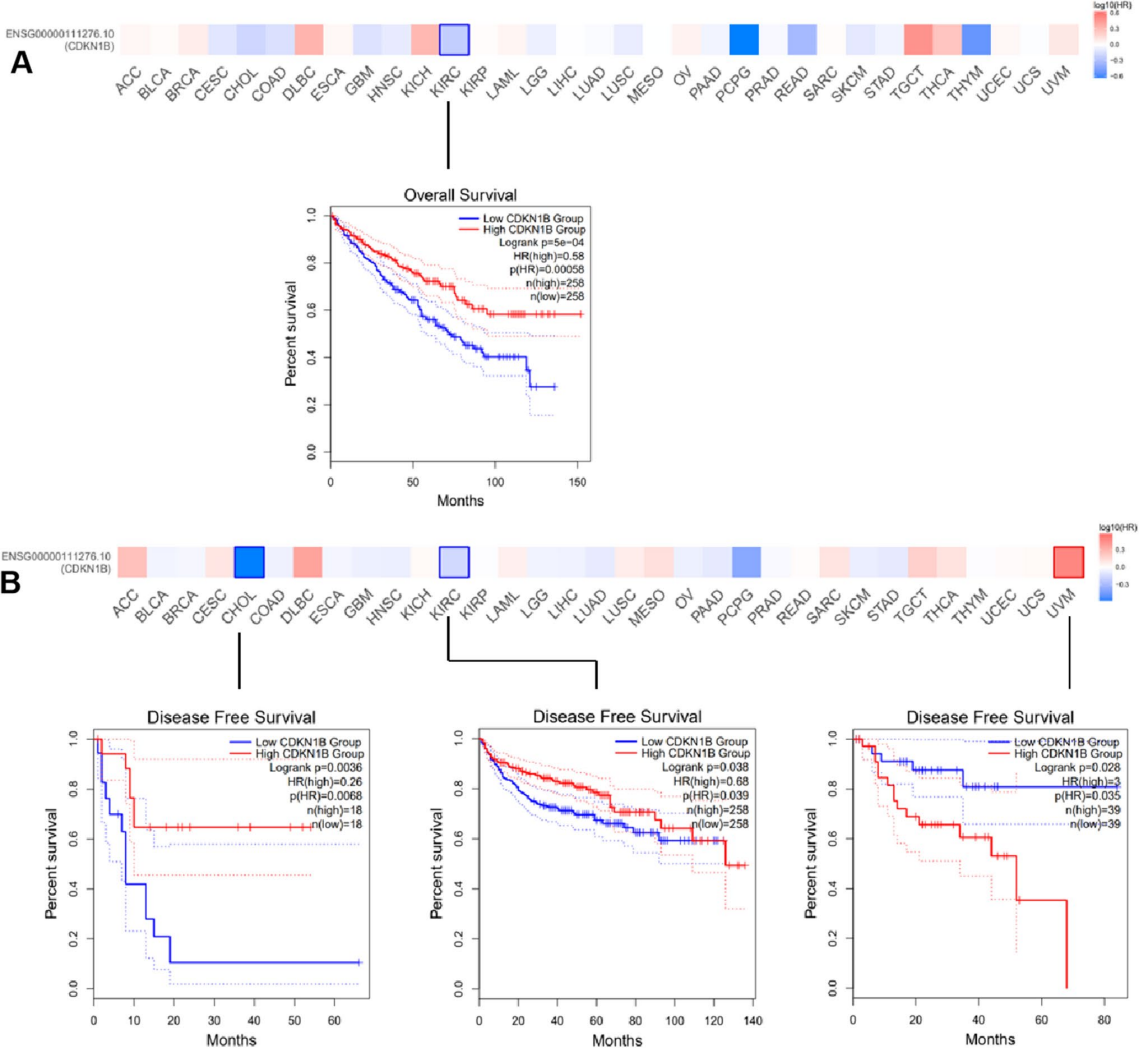
Correlation between CDKN1B gene expression and survival prognosis of cancers in TCGA. The TCGA dataset of various cancers was analyzed by CDKN1B gene expression using the GEPIA2 tool for overall survival (**A**) and disease-free survival (**B**) analysis. Positive results are shown for the Kaplan–Meier curves and the survival map

The cases were split into high- and low-expression groups according to the levels of CDKN1B expression, and the association between CDKN1B expression level and the prognosis of the patients with malignancies was then examined. According to the TCGA research, CDKN1B high-expression groups were linked to high odds of overall survival in KIRC tumors (Fig. 2A), as well as high odds of DFS in CHOL and KIRC cases. However, a high DFS prognosis for uveal melanoma (UVM) was associated with a low-expression group of the CDKN1B gene (Fig. 2B).

### Genetic alteration analysis

**Fig. 3:**
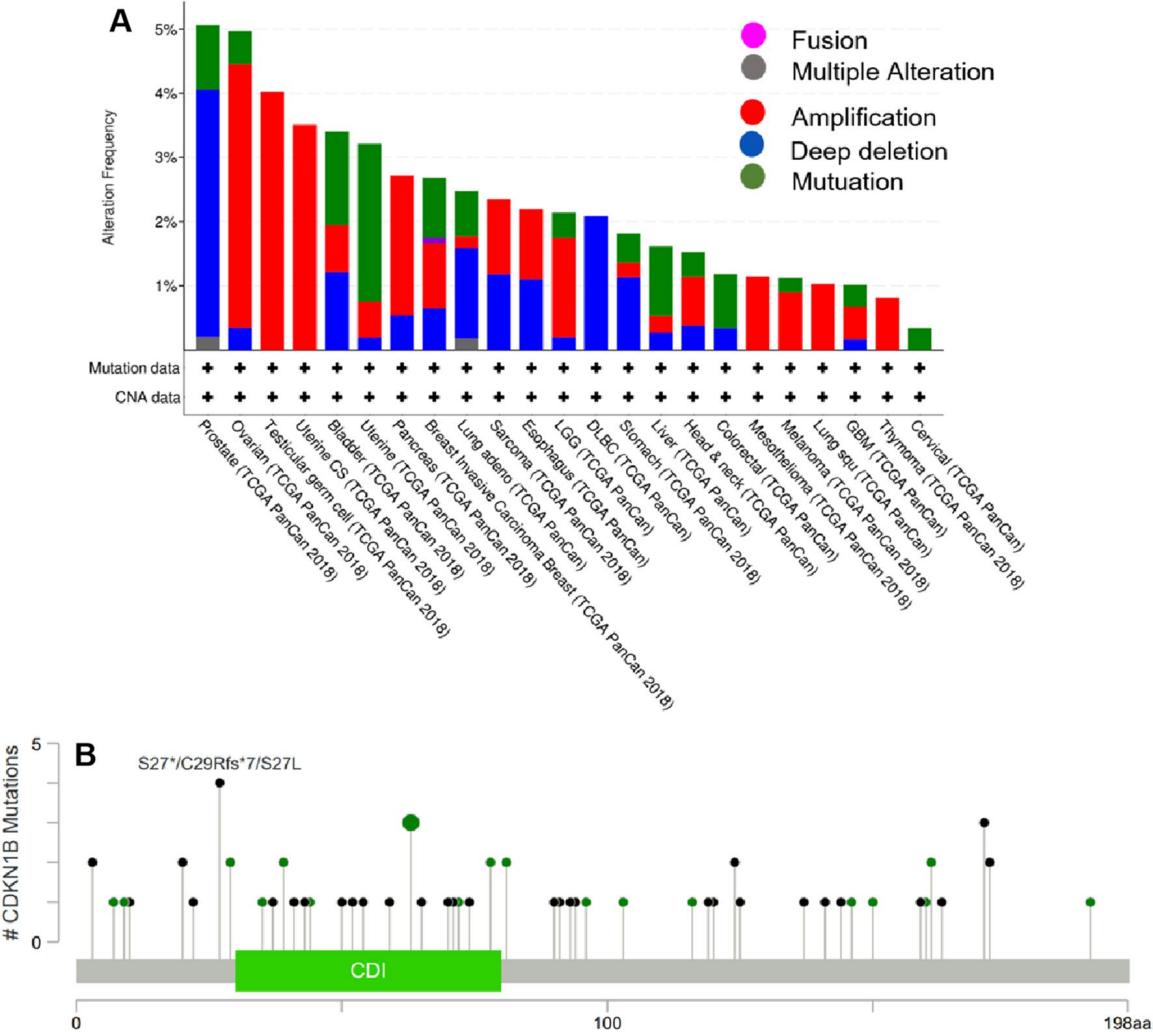
Mutation feature of CDKN1B in different tumors of TCGA. Using the cBioPortal program, we examined the mutation characteristics of CDKN1B in TCGA cancers. The frequency of the alteration is shown together with the mutation site (**B**) and type (**A**)

The genetic CDKN1B alteration in diverse tumor samples from the TCGA cohorts was presented in Fig. 3. The majority of CDKN1B mutations (> 3%) were found in uterine cancers with “mutation” as the predominant subtype in patients. In TGCT patients, “amplification”-type CNA predominated, which indicated a change (4% frequency) in CDKN1B copy number loss (Fig. 3a). The types, locations, and case counts of CDKN1B genetic changes were shown in Fig. 3b. A truncating mutation of CDKN1B was the primary type of genetic alteration, with Q65Rfs*6 alteration detected in 1 case of BRCA and Q65Rfs*60 alteration detected in one case of PRAD (Fig. 3B), which caused a frameshift mutation of the CDKN1B gene, translation from Q (glutamine) to R (arginine) at 65 site of CDKN1B protein, subsequently followed by CDKN1B protein truncation.

### Infiltration analysis of cancer-associated fibroblasts

**Fig. 4:**
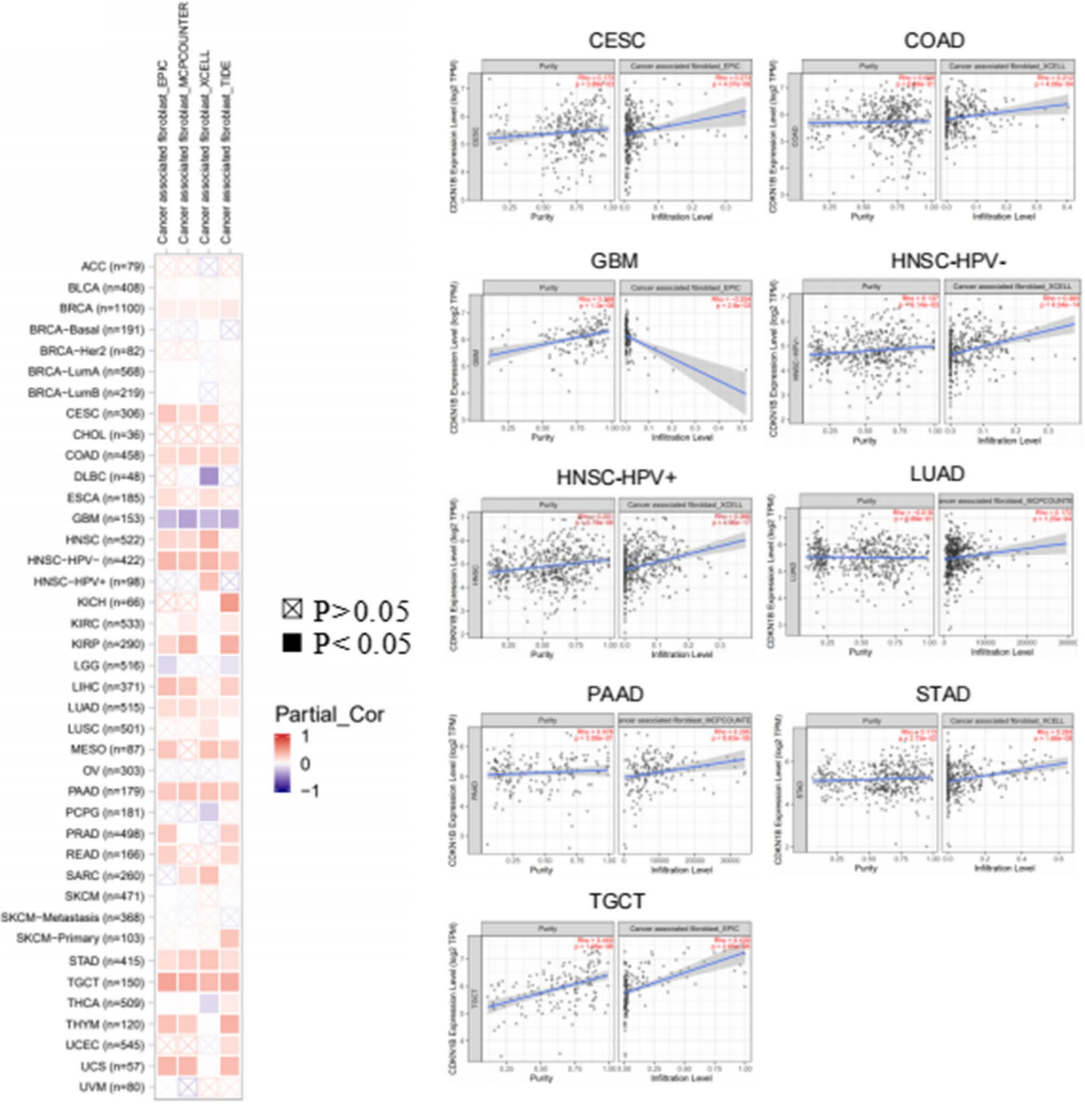
Correlation analysis between CDKN1B expression and immune infiltration of cancer-associated fibroblasts. Different algorithms were used to investigate the potential relationship between CDKN1B gene expression and cancer-associated fibroblasts infiltration across all types of cancer in TCGA

CAFs are crucial components of the tumor microenvironment (TME) and have a direct impact on how cancer starts, develops, or spreads. It has been suggested that cancer-associated fibroblasts in the stroma of the TME have a role in controlling how different tumor-infiltrating cells operate. Here, we utilized several algorithms, such as XCELL, EPIC and MCPCOUNTER, to investigate the underlying relationship between the amount of cancer-associated fibroblast infiltration and CDKN1B expression in various TCGA tumor types. The findings revealed that CDKN1B expression was statistically negatively correlated with GBM but statistically positively correlated with the estimated infiltration values of CAFs from TCGA, including CESC, COAD, HNSC, HNSC [HPV (human papillomavirus)], LUAD, PAAD, STAD, and TGCT (Fig. 4).

The scatterplot data of the above tumors generated through one algorithm were shown in Fig. 4. For instance, the MCPCOUNTER algorithm revealed a significant association between the level of CDKN1B expression in PAAD and the invasion of CAFs.

### Enrichment analysis of CDKN1B-related partners

**Fig. 5:**
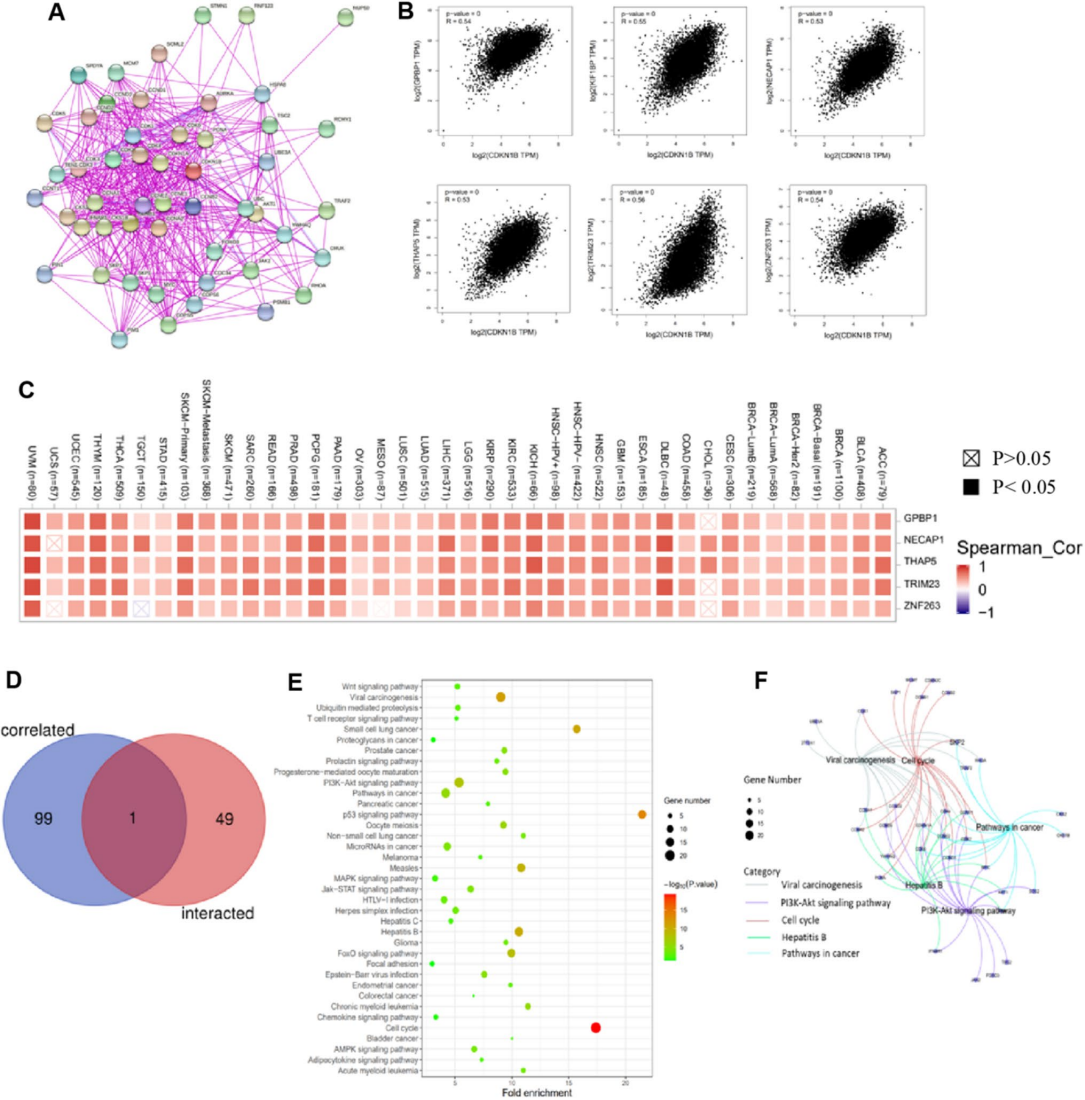
CDKN1B-related gene enrichment analysis. **A** Using the STRING tool, we first acquired the CDKN1B-binding proteins that have been experimentally confirmed to exist. **B** By employing the GEPIA2 method, we were also able to identify the top 100 CDKN1B-correlated genes in TCGA projects. We next examined the expression correlation between CDKN1B and a few target genes, such as GPBP1, KIF1BP, NECAP1, THAP5, TRIM23, and ZNF263, using these genes as our targets. **C** The relevant heatmap information for the specific cancer kinds is shown. **D** CDKN1B-binding and associated genes underwent an intersection analysis. **E** A KEGG pathway analysis was carried out using the genes that bind to and interact with CDKN1B. **F** The cnetplot for the GO analysis’ molecular function data is displayed

The CDKN1B-binding proteins and associated genes were screened for a pathway enrichment analysis in order to further investigate the probable mechanism of the CDKN1B gene in carcinogenesis. These proteins’ interaction network was shown in Fig. 5A. The GEPIA2 program identified the top one hundred genes that linked with CDKN1B expression. As displayed in Fig. 5B, the CDKN1B level was positively associated with that of the GC-rich promoter binding protein 1 (GPBP1), kinesin family member 1 binding protein (KIF1BP), adaptin ear-binding coat-associated protein 1 (NECAP1), thanatos-associated domain containing 5 (THAP5), tripartite motif containing 23 (TRIM23) and zinc finger protein 263 (ZNF263) genes, respectively. Additionally, the heatmap data showed that CDKN1B was positively correlated with the aforementioned six genes in the majority of the specific tumor types (Fig. 5C). Additionally, the intersection analysis of the two groups revealed one member with CCNT1 as a common member (Fig. 5D).

The two datasets from KEGG and GO were pooled for gene enrichment studies [28, 29]. The KEGG data revealed, as displayed in Fig. 5E, that “cell cycle” and “PI3K–Akt pathway” might be associated with the impact of CDKN1B on tumor pathogenesis [30]. According to the results from the GO enrichment study, the majority of these genes were connected to cellular biology or cellular biology pathways, such as the cell cycle, PI3K–Akt pathway, cell cycle, viral carcinogenesis, and cancer pathways (Fig. 5F). Additionally, the six CDKN1B-related partners’s mRNA levels were validated by PCR, and the increased mRNA levels were shown in Figure S1(A–F) in CT compared to PCT.

### Expression of CDKN1B in cancer tissues and adjacent tissues

**Fig. 6:**
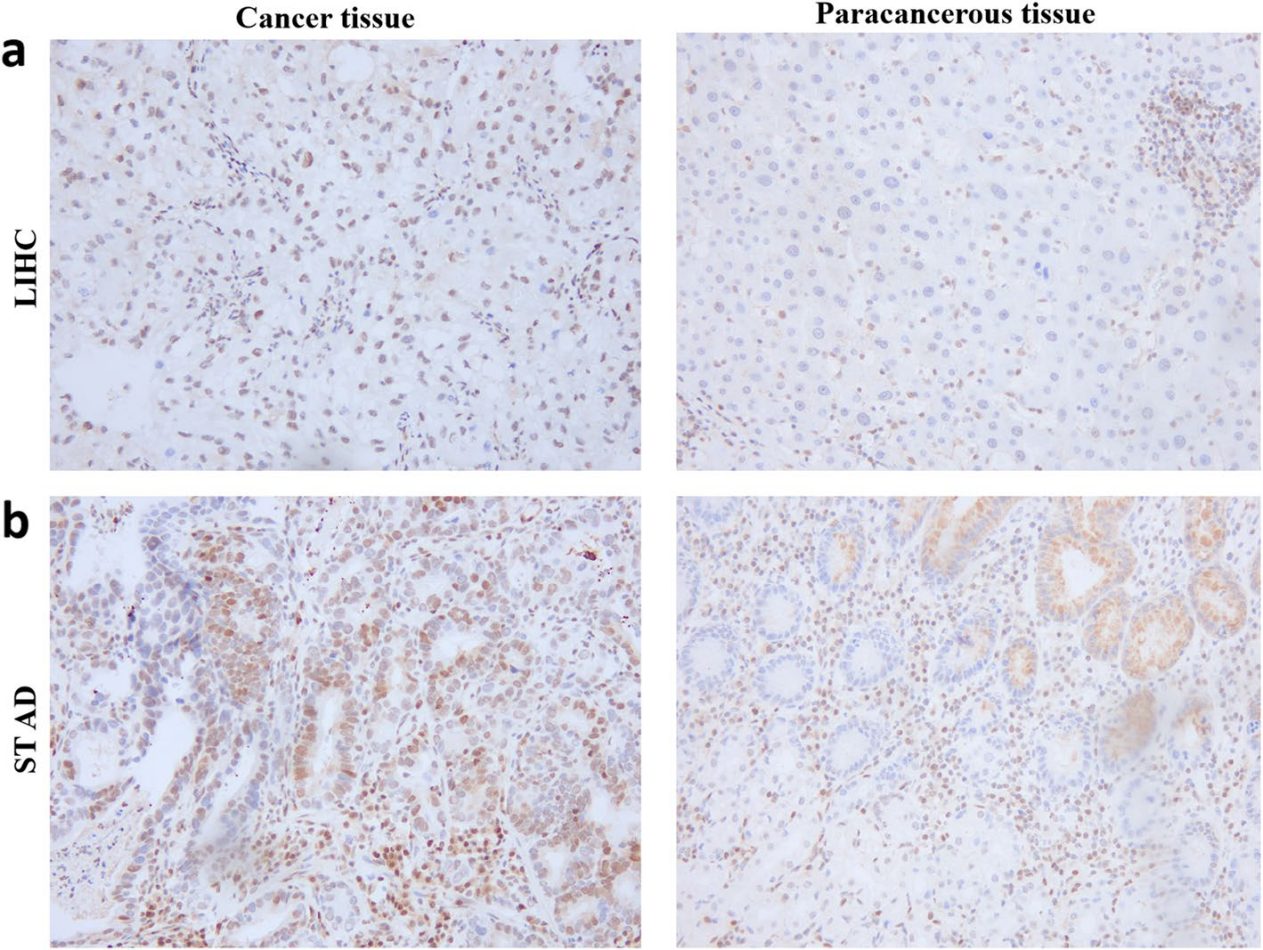
Expression of CDKN1B in cancer tissues and paracancerous tissues. Immunohistochemical (IHC) analysis showed that CDKN1B was highly expressed in LIHC (**a**) and STAD (**b**) compared with paracancerous tissues. LIHC (liver hepatocellular carcinoma); STAD (stomach adenocarcinoma). Positive staining of cancer cells is indicated by dark purple

IHC was performed to examine the expression of CDKN1B in STAD (Fig. 6a), LIHC (Fig. 6b), and the surrounding normal tissues in order to confirm the variances in CDKN1B expression between cancer tissues as well as surrounding tissues in the databases. The results displayed that CDKN1B expression was significantly expanded in the above cancer tissues (Fig. 6). The results match those discovered in the database. Additionally, the mRNA levels of the CDKN1B gene were validated by real-time PCR, which were significantly increased in CT compared to PCT, as shown in Figure S1(A–G).

## Discussion

It is well-known that CDKN1B is a tumor-inhibiting factor that encodes p27^kip1^, a cyclin-dependent kinase inhibitor [13]. The CDKN1B protein is associated with various cellular biological processes, such as gene transcription regulation, the cell cycle and cell apoptosis [31–33]. In addition, the CDKN1B-encoded protein also is responsible for cellular immunity, which is mainly reflected in its influence on the cycle progression of T lymphocytes [32, 33]. The cell cycle, including the progression from G0 to G1 to S phase, and both positive and negative regulators both govern the proliferation of immune cells, including T cells that express CDKN1B [32]. CDKN1B is a major negative regulator of the cell cycle, and its degradation can promote the transition of CD8^+^ T cells from G1 to S phase after TCR stimulation [32, 33]. It is still unknown whether CDKN1B has a role in the development of a number of cancers. Here, we firstly used the TCGA, CPTAC and GEO datasets to analyze molecular characteristics of gene expression and genetic alterations of CDKN1B.

CDKN1B is highly expressed in most of cancers, which was also confirmed in STAD and LIHC by IHC in our study. However, for different tumors, CDKN1B gene survival prognostic analysis data had reached different conclusions. In this study, the significant link between CDKN1B expression and the overall survival prognosis of KIRC, as well as the disease-free survival prognosis of CHOL, KIRC and UVM were also observed via the GEPIA2 tool. These findings suggested that CDKN1B was involved in the development of human cancers, which might be considered to be biomarker for the prognosis of cancers. Research is mounting that CDKN1B alterations are crucial in the development and prognosis of malignancies [34, 35]. The samples from ovarian cancer, testicular cancer, and uterine carcinosarcoma revealed that “amplification” was the most prevalent kind of mutation. In PCa, the “deep deletion” mutation type was more prevalent. According to earlier studies, a range of immune cells that infiltrate tumors are modulated by CAFs [35]. Combining the feature with the level of CDKN1B expression in various tumors, it was discovered that CDKN1B expression was significantly correlated with estimated infiltration values of CAFs from TCGA tumors, including CESC, COAD, HNSC, HNSC with negative human papillomavirus (HPV−)], LUAD, PAAD, STAD, and TGCT, but negatively correlated with GBM [36]. These findings indicated a close connection between cancer types, CDKN1B, and CAFs. Furthermore, previous studies indicated that CAFs have an active role in regulating HNC cell survival and proliferation by delivering functional miR-196a from CAFs to tumor cells via exosomes [9].

The significance of CDKN1B in carcinogenesis is critical for cancer diagnosis and treatment. Six genes were chosen among the 100 CDKN1B-linked genes for scatter plot mapping, and the results, together with the heatmap data, demonstrated that they were positively correlated with CDKN1B expression. These data also revealed, by cross-analysis, that CCNT1 is a common member. Cyclin T1 (CCNT1) is a gene with 9 exons that can assemble into a complex of positive transcription elongation factor b (P-TEFb) and regulate a variety of biological processes, including transcription [37]. These inklings indicated that CCNT1 may be the focus of further research.

Our results indicated that the cell cycle and PI3K/Akt signal pathway were involved in CDKN1B’s effects on the tumorigenesis mechanism. A previous study showed that increased Skp2 activity in malignancies with RB1 deficiency or mutations suppressed CDKN1B expression and led to an unregulated cell cycle. Skp2 was a target for cancer treatment and was implicated in controlling CDKN1B expression [38]. Furthermore, several studies have demonstrated that osteosarcomas’ overexpression of miR-802 impedes the PI3K/AKT pathway by targeting CDKN1B [39]. These findings might provide a potential role for human cancers in future research directions.

Using KIRC as an example, CDKN1B levels in malignant tissues increased than those in control, healthy tissues. Furthermore, in KIRC, a high prognosis for overall survival and DFS was linked to increased CDKN1B expression. In addition, previous report had shown that FGF-2 could promote the proliferation of KIRC by regulating p27^kip1^ [40]. Therefore, the pan-cancer study of CDKN1B can both widen research perspectives and support previous results. However, there are several limitations, particularly other independent cohort and in vitro or in vivo studies need be carried out to verify these results.

## Conclusion

The primary function of CDKN1B as a cell cycle inhibitor protein is to prevent or suppress cell division. Numerous studies have demonstrated that CDKN1B’s effects on the cell cycle have a significant role in the formation of cancers. This study firstly provided a pan-cancer analysis of CDKN1B’s involvement in various malignancies. This analysis revealed a statistical relationship between CDKN1B expression and various tumor characteristics, including clinical prognosis, cell infiltration, and gene mutation. This relationship assisted in clarifying CDKN1B’s function in oncogenesis from the perspective of clinical tumor tissues and provided a potential therapeutic target for treating human cancers.

## Supplementary Information

Additional file1 (DOCX 58 KB)

## Data Availability

The datasets generated during and/or analyzed during the current study are available from the corresponding author on reasonable request.

## Abbreviations

AML: Acute myeloid leukemia
CAFs: Cancer-associated fibroblasts
CCNT1: Cyclin T1
CDK: Cyclin-dependent kinase
CDKN1B: Cyclin-dependent kinase inhibitor 1B
CDKNI: Cyclin-dependent kinase inhibitor
CHOL: Cholangiocarcinoma
CNA: Copy number alterations
CPTAC: Clinical Proteomic Tumor Analysis Consortium
CT: Cancer tissue
DAVID: Database for annotation, visualization, and integrated discovery
DFS: Disease-free survival
DLBC: Diffuse large B-cell lymphoma
ESCA: Esophageal carcinoma
GAPDH: Glyceraldehyde 3-phosphate dehydrogenase
GEO: Gene Expression Omnibus
GEPIA2: Gene Expression Profiling Interaction Analysis, Version 2
GPBP1: GC-rich promoter binding protein 1
GTEx: Genotype-tissue expression
HCC: Hepatocellular carcinoma
HNSC–HPV±: Head and neck squamous cell carcinoma–human papillomavirus±
IHC: Immunohistochemistry
IRS: Immunoreactive scoring
KEGG: Kyoto Encyclopedia of Genes and Genomes
KICH: Kidney chromophobe
KIF1BP: Kinesin family member 1 binding protein
Kip: Kinase inhibitory protein
KIRC: Kidney renal clear cell carcinoma
KIRP: Kidney renal papillary cell carcinoma
LGG: Lower grade glioma
LUAD: Lung adenocarcinoma
NECAP1: Adaptin ear-binding coat-associated protein 1
OS: Overall survival
OV: Ovarian serous cystadenocarcinoma
PCa: Prostate cancer
PCT: Paracancerous tissue
PRAD: Prostate adenocarcinoma
P-TEFb: Positive transcription elongation factor b
qPCR: Quantitative polymerase chain reaction
rAAV: Recombinant adeno-associated virus
RCC: Renal cell carcinoma
SKCM: Skin cutaneous melanoma
SKP1: S-phase kinase-associated protein 1
STAD: Stomach adenocarcinoma
TCGA: The Cancer Genome Atlas
TGCT: Testicular germ cell tumors
THAP5: Thanatos-associated domain containing 5
THCA: Thyroid carcinoma
THYM: Thymoma
TIMER2: Tumor Immune Assessment Resource, Version 2
TME: Tumor microenvironment
TRIM23: Tripartite motif containing 23
UALCAN: University of Alabama at Birmingham Cancer Data Analysis Portal
UCEC: Uterine corpus endometrial carcinoma
UCS: Uterine carcinosarcoma
UVM: Uveal melanoma
ZNF263: Zinc finger protein 263
