# Supplementary material for: A pan-cancer analysis for the oncogenic role of cyclin-dependent kinase inhibitor 1B in human cancers
Source: Discov Oncol. 2023 Jul 11;14:126. doi: 10.1007/s12672-023-00746-8 (PMC10335976; doi:10.1007/s12672-023-00746-8)
Supplement: Supplementary file 1 — Additional file1 (DOCX 58 KB) [file 12672_2023_746_MOESM1_ESM.docx]

**Additional data**

**
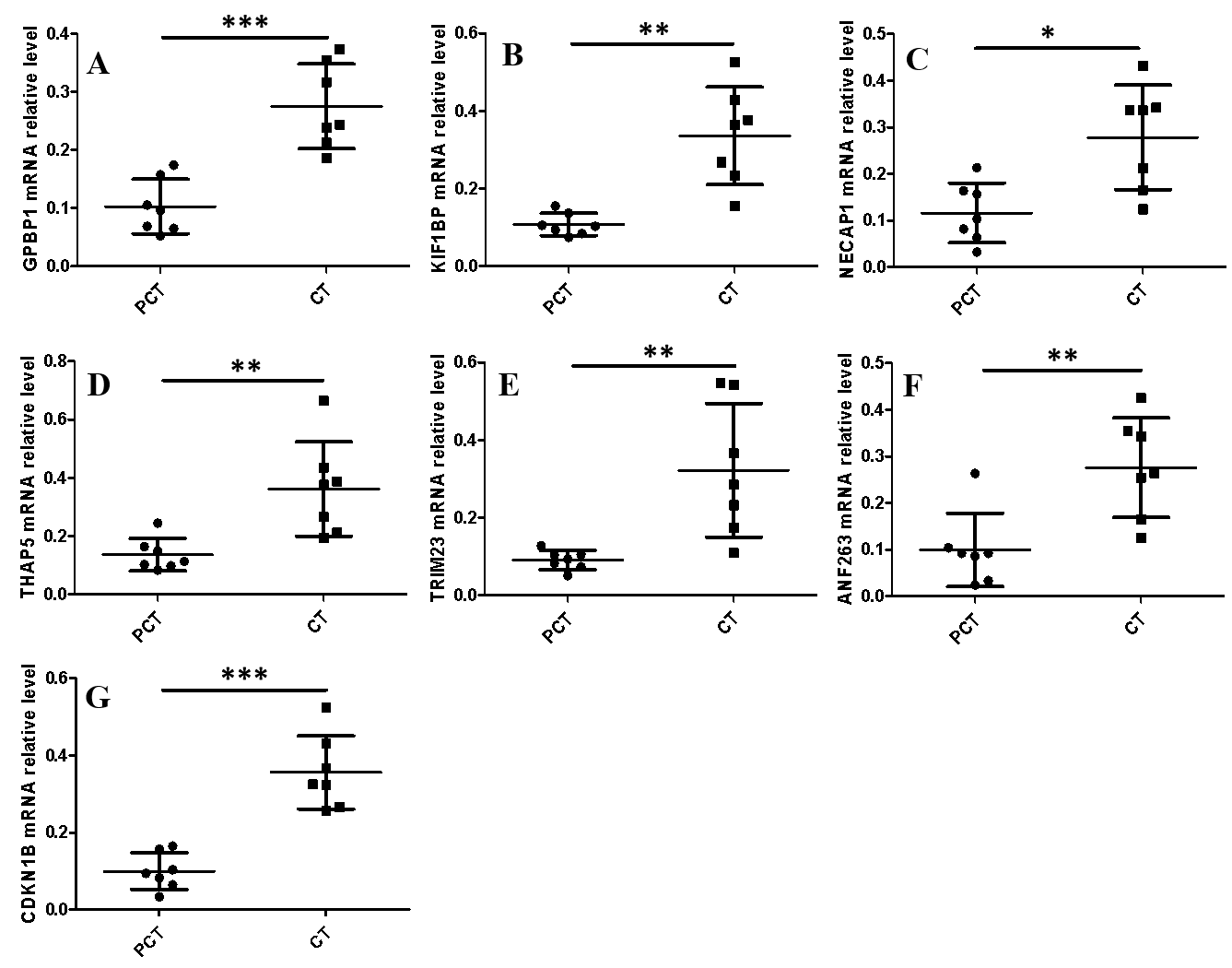
**

**Figure. S1. Relative mRNA expression levels of six CDKN1B-related partners in paracancerous tissue (PCT) and cancer tissue (CT) from seven human stomach adenocarcinoma (STAD).** (A) Relative GPBP1 (GC-rich promoter binding protein 1) mRNA levels (×10^-2^). (B) The KIF1BP (Kinesin family member 1 binding protein) mRNA relative levels (×10^-2^). (C) Relative NECAP1 (Adaptin ear-binding coat-associated protein 1) mRNA levels (×10^-2^). (D) Relative THAP5 (Thanatos-associated (THAP) domain containing 5) mRNA levels (×10^-2^). (E) Relative TRIM23 (tripartite motif containing 23) mRNA levels (×10^-2^). (F) Relative ZNF263 (zinc finger protein 263) mRNA levels (×10^-2^). (G) Relative CDKN1B (Cyclin-dependent kinase inhibitor 1B) mRNA levels (×10^-2^). Data represent the means ± SEM. *P< 0.05, **P< 0.01, ***P< 0.001.
